# *In Vitro* Corrosion and Cytocompatibility Properties of Nano-Whisker Hydroxyapatite Coating on Magnesium Alloy for Bone Tissue Engineering Applications

**DOI:** 10.3390/ijms16036113

**Published:** 2015-03-17

**Authors:** Huawei Yang, Xueyu Yan, Min Ling, Zuquan Xiong, Caiwen Ou, Wei Lu

**Affiliations:** 1 Departmentof Dentistry, Shanghai Tenth People’s Hospital, Tongji University, Shanghai 200072, China; E-Mails: yanghuawei2002@hotmail.com (H.Y.); yanxueyu2008@126.com (X.Y.); 2 Shanghai Key Laboratory of Development and Application for Metal-Functional Materials, School of Materials Science and Engineering, Tongji University, Shanghai 201804, China; E-Mail: luwis_1981@hotmail.com; 3 Department of Urology, Huashan Hospital, Fudan University, Shanghai 200031, China; 4 Department of Science and Technology, Southern Medical University, Guangzhou 510515, China; E-Mail: oucaiwennfy@gmail.com

**Keywords:** biodegradable Mg alloy, hydroxyapatite, hydrothermal process

## Abstract

We report here the successful fabrication of nano-whisker hydroxyapatite (nHA) coatings on Mg alloy by using a simple one-step hydrothermal process in aqueous solution. The nHA coating shows uniform structure and high crystallinity. Results indicate that nHA coating is promising for improving the *in vitro* corrosion and cytocompatibility properties of Mg-based implants and devices for bone tissue engineering. In addition, the simple hydrothermal deposition method used in the current study is also applicable to substrates with complex shapes or surface geometries.

## 1. Introduction

Bone tissue engineering is one of the most promising approaches to be used as alternative to the conventional autogenic or allogenic surgical techniques for bone tissue repair. Scaffold-based tissue engineering strategies involve the use of a biodegradable, porous scaffold that serves as structural template to fill the tissue lesion and to support cell-cell interactions and extracellular matrix (ECM) formation [1]. Different approaches have been employed to develop polymeric and composite scaffolds [1,2,3]. Magnesium and its alloys have been suggested as a revolutionary metallic biomaterial for the design of bone implant devices due to their excellent biocompatibility, mechanical properties (which are similar to natural bone) and degradability in a physiological environment [4,5,6,7]. The intended advantage of open porous scaffolds made of biodegradable magnesium is based on a fast biomaterial substitution by the regenerating bone from both sides: the rim and from within the scaffold. This design should lead to a faster replacement than a solid Mg implant which is replaced by surface corrosion. However, an open porous Mg scaffold has a huge surface which is prone to corrosion. The rapid *in vivo* corrosion rate of Magnesium-based scaffold has become a major obstacle to their applications in bone tissue engineering [8]. Therefore, corrosion control and slow initial corrosion is of utmost importance to avoid premature loss of strength and formation of local gas cavities. Elemental alloying is one option to reduce the corrosion rate of Mg alloys [9,10,11]. However, this may potentially lead to the introduction of toxic elements.

Surface modification with biocompatible protective coatings is an effective way to improve the corrosion property as well as the biocompatibility of Mg alloys [12,13,14,15,16]. Hydroxyapatite (HA, Ca_10_(PO_4_)_6_(OH)_2_), the main inorganic component of bone, is selected as the coating material in this study for its excellent biocompatibility, osteoconductivity and non-toxicity *in vivo*. In addition to control the corrosion of Mg alloys, nanostructured hydroxyapatite (nHA) coating can provide a desirable environment for bone tissue regeneration, as nHA mimics the nanostructure and chemistry of natural bone [17]. Natural bone is a nanostructured composite material composed of 70% nanostructured HA crystals of approximately 50 nm in length and 5 nm in diameter, and 22% organic Type I collagen fibers. Therefore, fabrication of nHA coatings on Mg alloys is not only a promising solution to delay the corrosion process, but also provide biomimetic interface for enhanced osteointegration and improved overall efficacy of orthopedic implants [6].

A one-step process using aqueous solutions consisting of non-toxic compounds is desirable for the formation of HA coating from the viewpoint of biocompatibility, production cost and environmental load. In the present study, we report the successful fabrication of nano-whisker HA (nHA) coatings on ZK60 (Mg-5.5wt%Zn-0.5wt%Zr) substrates by a one-step hydrothermal treatment in a novel aqueous solution with a pH of about 4.5. Keeping in mind the unique properties of nHA, the *in vitro* corrosion property and cytocompatibility of the nano-whisker HA coated ZK60 alloys are evaluated.

## 2. Results and Discussion

### 2.1. Formation of Hydroxyapatite (HA) on ZK60 Substrate

**Figure 1:**
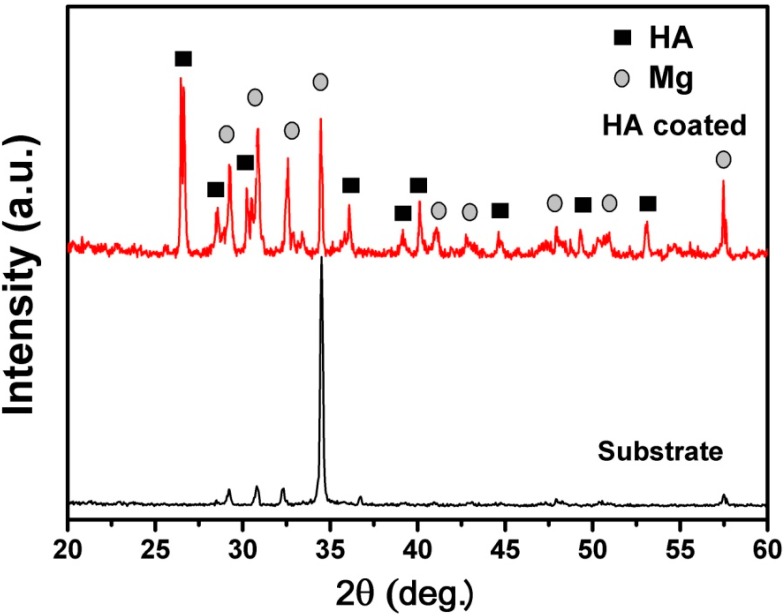
X-ray diffraction (XRD) patterns of uncoated and nano-whisker hydroxyapatite (nHA) coated ZK60 samples (a.u and deg. are the abbreviation of arbitrary unit and degree, respectively).

Figure 1 shows the X-ray diffraction (XRD) patterns of naked ZK60 substrate and coated ZK60 sample. Sharp diffraction peaks originating from HA phases were clearly observed in the patterns. The narrow full width at half maximum of the HA phase suggests that the crystallinity of HA is relatively high. Previously, it was reported to be difficult to form HA coatings on a magnesium surface in aqueous solutions because magnesium corrodes in solutions of pH below 11 [18,19,20]. Our results indicate that direct synthesis of HA on Mg alloy substrates using current method (pH = 4.5) is possible. In addition, crystallinity is an important concern on the HA coatings for implant application since low crystallinity accelerates the speed of dissolution of HA coatings in living body, causing the disappearance of coatings that bond to bone tissue at an early stage after implanting [21,22]. The HA coatings fabricated by current method are well crystallized, as confirmed with XRD analyses. High crystallinity is another advantage of the present method over most of the other coating approaches such as sol–gel coating, dip coating, thermal spraying, and sputter coating.

**Figure 2:**
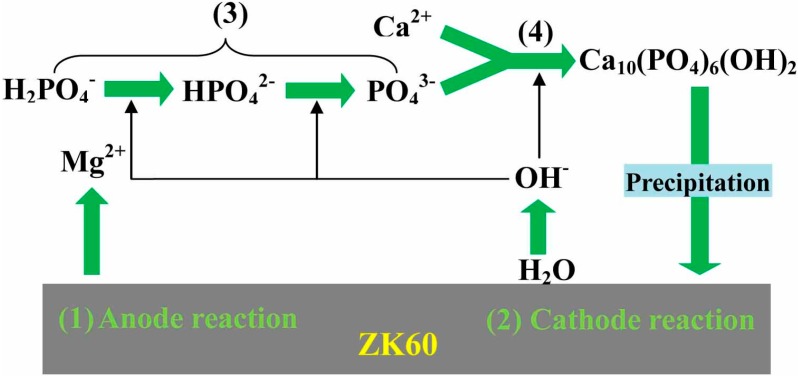
Schematic illustration of the reaction mechanism of calcium phosphate coatings on ZK60 substrate. (1) Anode reaction; (2) Cathode reaction; (3) Transformation of H_2_PO_4_^−^ to PO_4_^3−^; and (4) HA phase producing process.

The mechanism of HA deposition on the ZK60 alloy substrate can be illustrated by Figure 2. As the pH value of the treatment solution is about 4.5, the anodic dissolution of magnesium begins upon immersing the substrate in the coating solution, which leads to the release of Mg^2+^ ions, generation of bubbles and rapid increase of local pH on the magnesium surface during the initial stage of the treatment. This is explained by using the following reactions:

Mg → Mg^2+^ + 2e^−^ (1)

2H_2_O + 2e^−^ → 2OH^−^ + H_2_ (2)

The first reaction is the anodic dissolution of the magnesium metal and the source of the observed increase in magnesium ion. The second reaction is the reduction of water to give hydrogen gas, the observed bubbles, and hydroxyl ions which are responsible for the rise in pH value. The rapid corrosion of magnesium possibly raises the pH on the substrate surface to around 11 [23,24,25,26]. This rise in pH also has an effect on the phosphate species in the coating bath and enables the H_2_PO_4_^−^ to transform to PO_4_^3−^ through HPO_4_^2−^, as shown in the following reaction (3):

H_2_PO_4_^−^ → HPO_4_^2−^ → PO_4_^3−^ (3)

The appropriate local chemical environment in the vicinity of the surface contributes to the combination of the PO_4_^3−^ with Ca^2+^, producing a HA phase through the following reaction:

10Ca^2+^ + 6PO_4_^3−^ + 2OH^−^ → Ca_10_(PO_4_)_6_(OH)_2_ (4)

The initially precipitated HA covered the surface, which behaves as a protective layer separating the magnesium substrate from the treatment solution. In the latter stage of the synthesis, not only growth of the initially precipitated HA but also nucleation of HA in bulk solution result in an increase in the amount of HA precipitated.

### 2.2. Microstructure of HA Coatings

**Figure 3:**
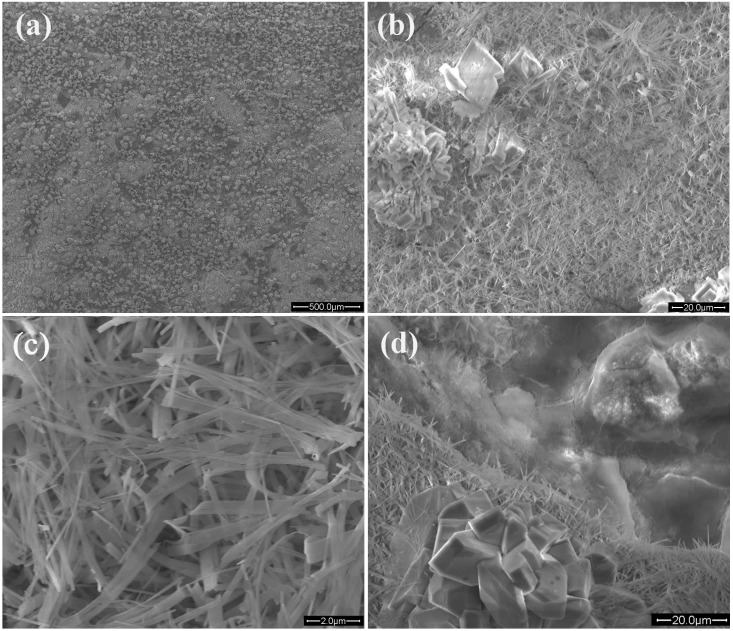
SEM images with different magnifications of nHA coatings on ZK60 substrate. (**a**) Integral morphology of the HA coatings; (**b**) Middle magnification SEM image; (**c**) High magnification SEM image; and (**d**) Bilayer structure of HA.

Figure 3 shows the SEM images with different magnification of the HA coatings on ZK60 alloy substrate. It can be seen clearly from Figure 3b,c that the HA coating is composed of coarse crystals and whisker-like crystals. The coarse crystals distribute randomly on the layer of whisker-like crystals, which covers the substrate uniformly and densely. It can be estimated from the SEM image that the size of coarse crystals is about 20–50 μm while that of whisker-like crystals is about 100–300 nm in diameter and about 3–10 μm in length. Whisker-like HA crystals have been shown to enhance bonding between implants and bone at the early stage of implantation [27]. In addition, it can be obviously observed from Figure 3d that the coatings are composed of two layers: outer layer with randomly distributed coarse crystals and densely covered nano whiskers: inner layer with dense dome-like structure. Based on the SEM image and XRD results, it can be expected that the dense and uniform coatings with single phase of HA could be formed on magnesium alloy substrates. A uniform, integral and crack-free coating is always desired in biomedical implants to enhance the coating-implant adherence. The HA coatings produced by hydrothermal deposition in current study promisingly meet this criteria. As an example, the low magnification SEM images in Figure 3a show the integral morphology of the HA coatings. The HA coatings are dense, uniform and without any observable holes or cracks which inevitably occur in the biomimetic process and electrochemical deposition. [5,14,15,28] As a comparison, an experiment using biomimetic deposition to grow CaP coatings shows that serious cracks formed in the coatings, as seen in [15,16].

### 2.3. In Vitro Corrosion Property

**Figure 4:**
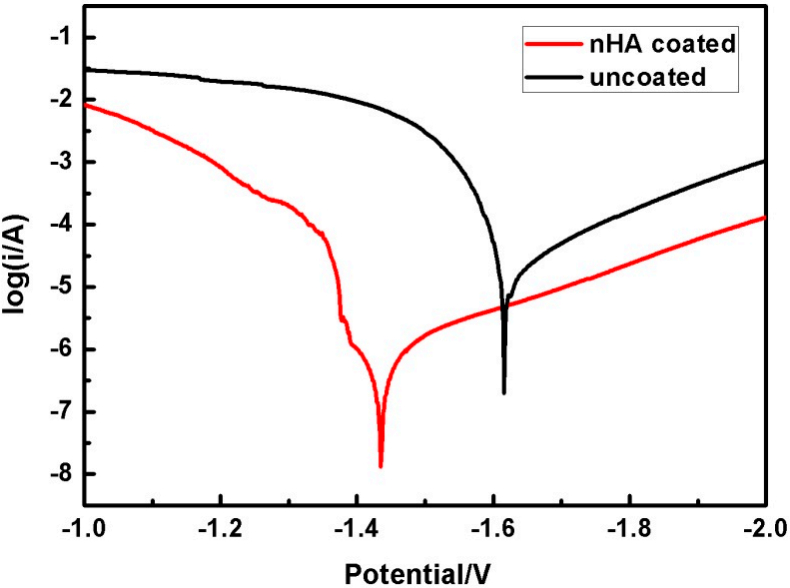
Potentiodynamic polarization curves of uncoated and nHA coated ZK60 samples.

Figure 4 shows potentiodynamic polarization curves of uncoated and nHA coated ZK60 alloys. The corresponding electrochemical data derived directly by Tafel extrapolation method are summarized in Table 1. Compared with uncoated ZK60, the *E*_corr_ values of the nHA coated ZK60 shift towards noble direction by 181 mV. Meanwhile, the corrosion current density *i*_corr_ is also decreased from 35.39 to 0.25 µA/cm^2^ (more than 2 orders) with the addition of the nHA coating. The result indicates that the corrosion resistance of the ZK60 alloy is significantly improved by the nHA coating.

**Table 1:** Electrochemical data extracted from the polarization curves.

Sample	*E_corr_* (mV)	*i_corr_* (µA/cm^2^)
Uncoated ZK60	*−*1666	35.39
nHA coated ZK60	*−*1485	0.25
AZ91 *	*−*1713	65.7

* The electrochemical data for AZ91 alloy are taken from the literature reported by Kannan and Raman [29].

**Figure 5:**
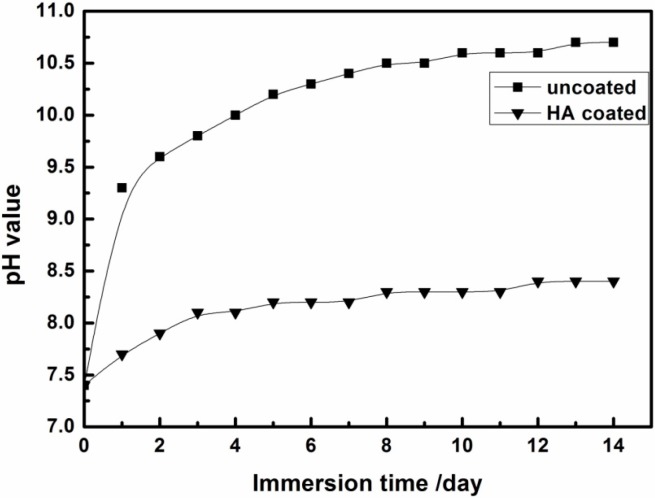
Variation of pH value of immersion solution at different immersion time of uncoated and nHA coated ZK60 samples.

To examine the long-term corrosion behavior, the *in vitro* corrosion behavior of the nHA coated and uncoated ZK60 samples were evaluated by immersion test in SBF. The corrosion rate of Mg alloys during the early stage of implantation plays a critical role in the initial surrounding tissue response. If the initial corrosion rate of Mg-based implants was too fast, osteolysis would occur, thus adversely affecting the regeneration of bone tissue [30]. Therefore, it is critical to control and decrease the initial corrosion rate of Mg-based implants. In the immersion test, few hydrogen bubbles appear on the surface of the nHA coated ZK60 alloy during the first several minutes. However, at the beginning of immersion, large numbers of hydrogen bubbles are evidently observed arising from the surface of the uncoated ZK60 substrate due to the reaction of the substrate with the corrosive electrolyte. During the whole immersion process, relatively rapid generation of bubbles from uncoated ZK60 substrate is observed, indicating a faster hydrogen evolution rate than that of the nHA coated substrate. Figure 5 shows the variation of the pH value of SBF solution at different immersion time. The uncoated ZK60 alloy shows a higher pH increase compared to the nHA coated sample. Furthermore, it can be seen that the pH values of the solution for uncoated and coated samples increase with different slopes by increasing immersion duration. At the first day, the pH for the uncoated sample increases from 7.4 to 9 while the pH for coated samples increase only about 0.3–0.6. After 2 days of immersion, the variability of the pH value of both immersion solutions slows down and the pH value tends to be stable over longer immersion time. After 2 weeks of immersion, the pH value for the uncoated sample reaches to about 10.7 while that for HA-coated samples are around 8.3, which is much lower than that of the uncoated samples. Therefore, it can be concluded that the samples coated with nHA have a better corrosion resistance than the uncoated sample.

Generally, corrosion behavior of Mg alloy is correlated to their microstructures. Mg is rather active in aqueous medium and dissolves according to the following reaction, which is derived from reaction (1) and (2):

Mg + 2H_2_O→Mg^2+^ + 2OH^−^ + H_2_ (5)

Consequently, in the early stage of immersion, the dissolution of magnesium leads to the release of OH^−^, resulting in the increase of pH value of SBF, and thus the change of pH value with immersion time can be used to evaluate the corrosion behavior of magnesium. With the accumulation of Mg^2+^ in SBF, Mg^2+^ would react with OH^−^ to form Mg(OH)_2_ which may precipitate on sample surface when reaching to its saturation. With increasing immersion time, the newly formed Mg^2+^, OH^−^ and Mg(OH)_2_ precipitates would reach a dynamic equilibrium, leading to a relatively stable pH in SBF.

**Figure 6:**
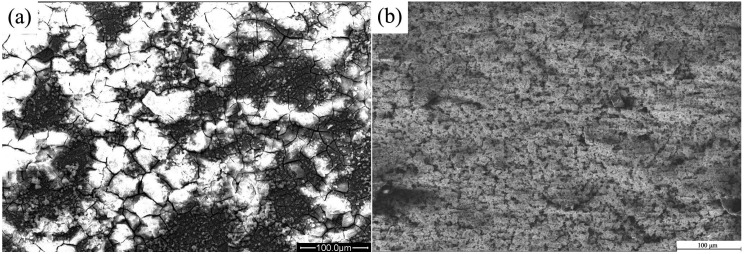
Surface morphologies of uncoated and nHA coated ZK60 samples after immersion test for 7 days. (**a**) Uncoated ZK60; and (**b**) nHA coated sample.

Figure 6 displays the surface morphologies of the samples after immersion test for 7 days. The uncoated ZK60 sample presents network-like cracks due to corrosion. The uncoated ZK60 (Figure 6a) corrodes seriously and a lot of white precipitates are formed on the surface. The precipitates are mainly composed of Ca, P, O, C, Mg and Zn elements, suggesting the formation of bone-like apatite on the surface. Figure 6b shows the corrosion morphology of nHA coated sample after 7 days of immersion. Compared with the nHA coated sample before immersion, no significant changes in the morphology are observed. The coatings still cover the ZK60 substrate completely and can provide protection for the substrate from corrosion. No visible cracks and evident corrosion phenomenon can be observed on the HA coated sample after immersion testing, in comparison with the uncoated alloy, suggesting that HA coating exhibits very good corrosion resistance.

### 2.4. In Vitro Cytocompatibility

**Figure 7:**
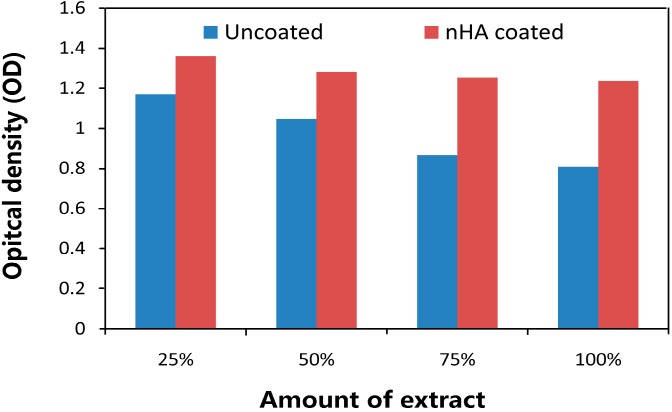
Cell viability cultured in individual extraction mediums of uncoated and nHA coated ZK60 samples.

Figure 7 shows the L-929 cell viability cultured in individual extraction mediums of uncoated and nHA coated ZK60 alloy for 3 days. After 3 days of culture, a decrease of absorption is observed for both samples. MTT tests indicate that the absorption for uncoated ZK60 are significantly lower than that for nHA coated sample at all amounts of extract, especially at 75% and 100%, suggesting that the cytocompatibility of the substrate are improved by the coatings. From the analysis of the above results, it can be concluded that the coated ZK60 alloy has excellent biological cellular responses *in vitro*, as well as enhanced bioactivity, due to the nanostructured nature and high degree of crystallinity of HA coatings. Previous *in vitro* cytotoxicity studies on magnesium alloys have suggested that, a lower corrosion resistance leads to a higher pH value caused by corrosion, which finally reduces cell viability [31]. It is expected that, with enhanced corrosion resistance by the coating, the coated ZK60 will have higher cytocompatibility than the uncoated one. This expectation is demonstrated by our MTT results. The results of electrochemical test and immersion test agree well with each other and they are exactly the opposite trend of MTT results, demonstrating the improvement of corrosion resistance and cytocompatibility of ZK60 by nHA coating.

Although the corrosion behavior of nHA coated Zk60 alloy is described in details in this study, as well as the coating properties, more investigations of biochemical and biological nature are required to completely access its full potential for the desired application.

## 3. Experimentals

The ZK60 alloy substrates were polished with SiC papers up to 2000 grit, ultrasonically cleaned in ethanol and dried in air. For the preparation of HA coating, aqueous solution of 100 mL containing 0.05 M Ca(NO_3_)_2_·4H_2_O and 0.03 M NaH_2_PO_4_·2H_2_O was prepared by dissolving analytical grade reagents Ca(NO_3_)_2_·4H_2_O and NaH_2_PO_4_·2H_2_O in deionized water at room temperature. The autoclave used in this experiment is a home-made device composed of a stainless steel autoclave with a Teflon liner. Subsequently, ZK60 substrates were put into the autoclave, heated to 140 °C by an oven, and then kept at this temperature for 2 h. The non-coated Mg substrates were used as the control. Surface morphology and crystal structures of the nHA coated and non-coated Mg samples were characterized using scanning electron microscopy (SEM, Quanta 200 FEG, FEI Company, Hillsboro, OR, USA) and X-ray diffraction (XRD, D/max2550, Rigaku, Japan), respectively. The conventional three electrode cell was used for the electrochemical measurements. Immersion tests were carried out in home-made simulated body fluid (SBF solution at 37 ± 0.5 °C. The pH value of SBF solution was monitored with a pH meter (Mettler-Toledo, Switzerland). Murine fibroblast L-929 cell (purchased from cell bank of Chinese Academy of Sciences, Shanghai, China) was adopted to evaluate the cytocompatibility of the samples.

## 4. Conclusions

In summary, HA coatings with nano-whisker structure were successfully developed on ZK60 substrates by a simple hydrothermal deposition method. The deposition includes several reactions to form coating of HA phase. The coatings fabricated by current method show uniform structure and high crystallinity. In comparison to the uncoated ZK60 sample, the nHA coated ZK60 sample shows significantly improved corrosion resistance and cytocompatibility properties. Results suggest that nHA coatings are promising for applications in the field of bioactive surface modification for Mg-based implants and devices of bone tissue engineering.

In addition, the simple hydrothermal deposition method used in current study is also applicable to substrates with complex shapes or surface geometries so that little restrictions on implant shapes are required in the coating process.
